# Annual Address

**Published:** 1878-04

**Authors:** A. O. Rawles


### THE DENTAL REGISTER.

VOL. XXXII.] **APRIL 1878.** NO. 4.

### ANNUAL ADDRESS.

*To the Graduating Class in the Ohio Dental College, March 6th, 1878.*

BY A. O. RAWLS, D. D. S.

In behalf of the president and faculty of the Ohio College of Dental Surgery, I have the pleasure of greeting you as you take your exit from the imposed duties of collegiate life, and at the same time to welcome you most heartily into the ranks of a profession not ashamed of her progress in the past, nor yet fearful of her honors in the future.

The zeal with which you entered upon and continued the performance of duties appertaining to college regulations is, we trust, a faithful indication that active, earnest labor, will characterize your entire professional life. And now that you are enlisted in the cause of education by becoming sharers in its inestimable endowments, let me insist that you lessen not your energies in this direction, but join hands with those who have burned low the midnight oil that you might be benefited, and assist the profession of your choice, in solving the problem of education for your mutual good, the good of others entering the same gates you have entered, and in behalf of common humanity.

For the purpose of stimulating any desire you possess to assist in this work, I shall digress somewhat from the usual method of giving advice for your future guidance, and call your attention to the subject of dental education.

Viewed in its general application, the problem of education holds a position transcending in importance, complexity and perplexity all momentous questions of ancient and modern times, and while its absolute solution is beyond the possibility of human power in this or future generation, its comparative solution challenges a world's admiration, as well as centuries of unceasing labor. That the problem is being simplified and solved in accordance with the various demands arising from the constantly changing conditions of the world's history no one can justly doubt, and, as little by little we discover its solution as applied to art, science, and the various industries, so in like manner with each successive step in advance, are we the better prepared to meet new phases of this problem, and in turn, present to our successors a more perfect mode of education.

In consequence of the various and diverse opinions held by our leading educators, relative to the proper mode of instructing students, it has been and still continues a matter of impossibility to secure any action bordering upon unity of design in the management of our educational institutions, and prominent among the questions at issue, which have served no little toward animating and intensifying expressions opposed to the true interests of every college and the profession, are those concerning the relative position of dentistry to medicine, and the propriety of attaching dental departments to regularly incorporated medical colleges.

It is not a part of my intention in this address, to enter very deeply upon the merits of this question, as it refers to colleges alone, suffice it to state that I am strictly and perhaps irreconcilably opposed to any scheme by or through which dentistry would in any wise compromise its struggle for independence, or, in other words, would lose its individuality, and be swung as a tail to the kite of another profession. The medical profession do not as a rule, desire that such should be the case—not on account of the tail being too light, neither from fear of its assuming proportions, which would entitle it to the kite's position, and reduce the medical profession to the extremity of a tail, but more likely for the reason of indifference founded upon a belief that it can not materially effect their condition, no matter how the relation exists. It is true that a very small minority of the medical profession insists that we should be under their educational care, but none of this number happen to be leaders of opinion in their calling. It is of equal truth that there are but few of our profession coinciding with them, and these few happen to be pseudo leaders of reformative dental education.

Now we do not insist that you should draw comparisons, for you are surely aware that they are sometimes odious, and the above if scrutinized too closely might appear extremely so.

The great mass of our profession desire independence in our system of education as well as in our professional standing, not, however, for a moment thinking that it is possible to be independent of the assistance tendered us at the hands of educated labor, resulting from investigation to meet other ends, but independent in the sense that other professions are independent of each other, and yet dependent one upon the other, for such portions of acquirement in one as will apply to the exigences of the other.

In our relation to science and art, in fact to everything aid ing our development to use the language of an eminent divine, we might be compared to a wheel when the spokes are all in place, and the circle perfect, it runs without a jar, but remove a segment from the circle, and it shivers at every turn; so in like manner are we dependent more or less upon each segment composing the circle of our educational relations; let any one be removed, and at every step will be felt more and more sensibly the shock occasioned by the break. In this manner then, we frankly and with gratitude acknowledge favors at the hands of medicine, and are willing to grant that a portion of our calling might with propriety be termed a specialty of medicine, but as an entirety, it surely stands out separate and apart in the great wheel, touching hands only that it may give, and with open palms that it may receive the precious gems of truth, constantly passing from segment to segment throughout the circle.

One of the many obstacles to a more advanced standard of dental education, is the too great number of dental schools, as compared with the demands of our profession, and, as an evidence that these colleges are in excess of the demands, we have only to note the zeal and eagerness characterizing their annual pursuit for material to educate .That they are well supplied with teachers thoroughly acquainted with both the theory and practice of their several departments, no one will doubt, but by accepting the statement that they are thus cognizant of principles and practice, we make easy the inference to at least a casual observer, that they are also capable of teaching with success that which they have so thoroughly mastered, and yet should such an inference be always drawn, or in other words, does it always follow that accomplishments can best be taught by those the most accomplished. On the contrary, is it not of rather rare occurrence, that the ability to impart information is blended equally with the knowledge of that to be imparted? Are there not many members of the profession, who, though perfectly conversant with every requirement of dentistry, are nevertheless barely fitted for the position of tutors to a private student, and again is it not true that many who are entirely adequate to the task of imparting knowledge to particularly qualified students or classes of students, are notwithstanding wholly devoid of that peculiar attainment, which renders its possessor equal to the labor of adapting his mode of instruction to the wants of a varied class. In support of at least a part of these statements, we have but to recount the number of times during our professional career we have held to doubtful opinions with such dogmatic ardor, as to render our teachings dangerously unfit for the ears of confiding students. Putting it somewhat plainer, we have at times tumbled bag and baggage into a rut, have been hobby riders, too intent on the singleness of our view to escape into the generous whole-some atmospheres of reason and progress, and too wise in our own estimations to seek or even desire the assistance of fellow co-laborers. But happily for us, and for all who are to share in the interest of our calling, these things are passing away. Our associations with their committees of investigation, our local societies, dental literature, and the results of individual zeal borne to our very doors from every direction warn us alike to be chary of hobbies, to keep our minds open to conviction of the truth, and our views prepared for criticisms.

It is a fact patent to all, that few of our writers on the subject of dental education ever refer to the matter, except as it concerns directly collegiate institutions, and their past or present discrepancies in modes of instruction, and if it were possible for colleges to have no mutual interests with the profession, that from time to time points young men to their doors, then indeed might it suffice to treat thus exclusively this subject.

But surely these colleges in common with the profession have a most decided interest in the matter of how best to secure and mould material for scholastic honors, and if we would advance our interest herein, desire to understand more correctly the character of difficulties opposed to real advancement, in our opinion no more appropriate course can be pursued, than that of beginning our investigations with the office student, tracing step by step the demands of his progress through the varied phases of so called educational life, till the time when he will bid adieu to his *alma mater*, and launch out into the troubled sea of individual responsibility.

Previous, however, to a consideration of the foregoing, it will doubtless be of interest to inquire into the matter of selecting young men of proper qualifications for entering upon the study of dentistry.

The importance of a judicious discrimination in the selection of applicants for studentship, is beyond question, and a mistake once made herein, ushers into existence the nucleus to a long and continuous line of mishaps, the out-growing evils of which will be felt more or less by all, but by none more than those who are laboring so earnestly in behalf of our educational interests. Under the most favorable circumstances we are liable to err in our judgment, concerning the qualifications of all applicants, this to, notwithstanding our honesty of purpose in making a choice; aside from this it is not in the nature of men to look at this, or for that matter any subject from the same stand point, consequently, results will vary accordingly, and yet it is evident that out of more general appreciation of the import of this special phase of our subject, a plan or system of rulings might be developed which would ameliorate very materially the present status of things.

The better class of dentists are, as a rule, particularly careful in the matter of receiving students. Neverthless, they not unfrequently fail to see clearly the acquirements and intentions of applicants. To you my young friends I speak more directly of the intentions of an applicant, and trust you will, if circumstances require, give them due consideration, for they sometimes carry as much force and will deserve as careful scrutiny, as will other requisites for study and practice.

There is another class of dentists, however, numbers of which seem totally ignorant or unmindful of the necessity and wisdom of judicious discrimination in reference to this subject. They are not in the least exacting, but on the contrary accept all applicants in their power to accommodate, regardless of their fitness for the labors before them. Little does it interest them, whether their choice be good, bad or indifferent, since in making it they were impelled by none other than mercenary motives.

Notwithstanding however, the evils resulting from dishonest intentions herein displayed, it might be considered a redeeming feature that out of this indiscriminate corps of students comes a few of our more cultured and zealous co-laborers, in view of this it would be natural for us to ask whether we should not render honor to whom honor is due.

Unquestionably this is our duty, but to whom say you the honor in this case is due? Surely not to the preceptor, who by mere chance and wrong intent made the selection. Nay! but rather to the student who fortunately possessed enough good sense, to scan correctly the view from his own stand point and rise superior to the indifferent surroundings which might otherwise have kept him upon a level with his so called tutor.

But as to the greater number of this latter class of promiscuous choosing, it could, in truth, be said that they are almost, if not entirely incompetent to select intelligently vocations suited to or in keeping with their real proclivities, and now the most pertinent question is, what can be done with them.

They must either be debarred from practice, educated aright, or their influence for evil will continue a subject of concern, affecting not alone the interest of their patients, but interfering with the system of education and to no little degree, baffling the efforts and desires of a fraternity, whose conscientious aims ever look to the welfare of its patrons and members. In order that we may be intelligently prepared for breaking down the barriers between our present standard of education, and that to which we would attain, there is no one thing probably of more importance than a recognition of the fact that in different communities we will be compelled to deal with very different orders of intellect. General education, even in this land of free schools is so unequal that anything comporting with the desires of one community would in all probability fall far short of the demands of another, and it is to this condition of our country's population, that we are undebted for much of the widespread charlatanism, against which we are now struggling, and while communities are thus contented with a low order of dentistry, so accordingly will students from such localities be governed in their preparation for practice.

To remedy this state of things we have recourse to but two modes of action, viz: By creating in the different parts of our country a demand for excellence, or by securing the enactment of state laws, compelling persons entering the profession to have or acquire the necessary qualifications before allowing them to practice.

We doubt not that much time and labor must be expended, before either of the foregoing ways could be opened or imposed, but since in some parts of our domain such restrictions have been placed upon the profession and thoroughness has become one of the principal passports to confidence and the establishment of practice, surely it would be unwise in us to doubt for a moment its feasibility throughout our land.

It is a matter of regret that so many of our number are either directly antagonistic or through stupid passivity are indirectly opposed to any movement, having for its end the advancement of our professional standing. Deplore it as we may, it is nevertheless too true to be questioned.

Yes here under the bright light of genius which has led dentistry out of the wilderness, and to its present position here under the glowing radiance of the nineteenth century with two centuries of our past progress to guide them, with colleges at their very doors, with the eyes of Hunter, of Bell, of Jourdain, of Fox and Harris, looking down upon them and the pleading interests of forty millions of people, demanding our undivided labors, these men stand battling against their best interest or sculk away in the gloom, that they may shun the labors which by all sense of honor, of justice and of humanity they are pledged to perform.

Why this is true of so many, is beyond our comprehension, but if we could look down into the hearts of a few, we would doubtless find there not opposition to progress *per se*, but a heart full of bitterness toward any plan, the organization or workings of which would open avenues of honor or preferment to any save themselves.

In view then of these and other hindrances, and that our efforts may not go unrewarded, it is surely apparent that the demands of the hour would urge upon us the necessity of a more united professional strength, and added to that a more thorough going individual activity; an activity too buoyant to rest contented with the ordinary labors of daily practice, and an union of strength such as will command respectful notice at the hands of our more intelligent legislators, and compel acquiesence upon the part of malcontents, who through stupidity stand in their own light.

When the consummation of these ends is assured, either through the demands of a well instructed popular mind, or as the result of legislative enactment, we will then have paved the way for entrance into the profession, of those who possess a proper appreciation of its requirements, and who are at least fairly qualified for the duties before them—indeed, those who are not so qualified will hardly desire an entrance.

Having stated our conviction, relative to the selection of students, we will now call attention to that phase of the subject next in order, viz: that of office instruction. There is evidently sufficient of importance connected with this to deserve more consideration at our hands than has heretofore been devoted to it, and we can not refrain from expressing the opinion that much injury has resulted to both student and college by a lack of compliance with its demands. Quintilius says, "Let him that is skilled in teaching, first of all, when a scholar is entrusted to his care, ascertain his ability and disposition." That is certainly good advice and full worthy of application by all who desire success in teaching. Those of my hearers who have had much experience in these matters, are aware of the fact that the same mode of instruction will not produce equal results upon different persons. This, for the obvious reason that no two persons, even of the same age, and having had similar advantages, are by nature alike in their habits, tendencies or qualifications. The inference to be drawn from this is both easy and natural, and to the effect that each one should be taught with reference and in a manner conforming to existing peculiarities.

The turn, or as oftener expressed, the bent of a student's mind, usually leads, if untrammeled, to study of certain branches of his calling in which he is the most desirous of becoming proficient, and generally speaking, his advance within the scope of such desires is much more rapid than would be the case were he studying in departments not strictly suitable to his tastes. There are some, however, whose mental proclivities are not so very marked in any special course, and yet they seem to seize readily any idea you may present, carry it out, slowly it may be, but to its conclusion within the bounds of the desired application, and in the meantime keep fairly up their investigations in collateral branches. The latter class is by all means the least troublesome to instruct; its members yield more readily to the labors imposed, and apparently become as much absorbed in one as in another department. Members of the former, those who are possessed of superior talent for the pursuit of knowledge within certain limited bounds, are much more difficult to teach principles which would aid them in securing the necssary acquirements in each of the several branches of dentistry.

In order then that the students may be perfected in the several lines of thought and varieties of labor it is clearly apparent that their peculiarities should be recognized by their teachers. We should note the student's abilities and preferences in certain directions; his lack of ability and want of concern in other matters, and wherein there existed a showing of weakness or inability, leave nothing undone which would in anywise contribute to interest his attention. It is true that a tenacious, self-sacrificing devotion to special order of study generally results in superior excellence in the line of labor pursued, and yet should we apply this to the study of special departments of dentistry, is it not of equal truth that such advance, if attained without proper consideration of relative subjects would hardly deserve the merit of true excellence.

If it were possible to separate the different branches of dentistry in a manner that would enable one to study and practice either branch successfully, and regardless of any knowledge appertaining to other departments, then indeed such a course might obtain, but since the specialties of this specialty are so intimately linked one with another, it becomes necessary to master them, or at least have a good general knowledge of all before we may hope to attain true excellence of either. It is doubtless the experience of colleges as well as private preceptors, that students long before they are fitted for such labors become so enamored with the operative department as regards more specially the process of filling teeth, that they are lost to all sense of the principles which should govern them therein, and which are of paramount importance to mere manipulative skill. That such things ultimately operate adversely to the student's interest no one can justly doubt, and the earlier these tendencies are checked the better will it be for all concerned.

In the office preparation of students, their future course of instruction in college should be continually in view, and day by day gradualy approached, thus enabling them when entering upon the duties of such collegiate course to avoid the necessity of reviewing studies belonging more properly to private pupilage.

The mode of instruction should be of such nature and so systematized that upon their entering college it would only be to renew their studies without any very material break in the former line of thought or investigation.

To say that these things can not be accomplished would be tantamount to a libel upon numbers of our preceptors who have for years past, time and again proved its effeciency beyond the shade of a doubt; but the question now is, how can we make this mode of office training the most general? How can we demand of each and every preceptor to pursue this or that course, or in what way may we effect the general application of these rules of graded systematic office instruction? Its consummation would indeed, be a foregone conclusion, were it in the possible power of the profession unaided by college requirements, but it is not in our power alone to consummate these ends. On the contrary, our endeavors to render assistance to both student and college, are, we regret to say, made more or less inoperative or at least not encouraged by the action of a few colleges relative to the qualifications necessary to the student's entrance therein. If we are not mistaken, however, most of our dental colleges are operating or did at one time operate in reference to these matters under the restrictions of certain rules and regulations, imposed by an act or actions of the National College Association, and as we understand the course agreed upon by the representatives of different institutions composing said association it is certainly commendable, and in keeping with the highest interests of the profession. But alas for the rectitude of collective action. The spirit of individual interest and aggrandizement has crept in, and where emudative agreement should have characterized the sentiments of each college toward its neighbor, too often there has existed selfish rivalry, which left unconsidered the true principles upon which they should labor.

It is this inconsistent, and we might add disrespectful, rivalry that has served to open some of our college gates for unqualified students thereby recognizing inexcusable ignorance as upon a level with labored excellence, discouraging a proper preparatory course by encouraging careless inability, and a desire upon the part of many students to accomplish all at a bound. I say many students, not all far be it from my thoughts to thus impugne the motives of all indiscrimately, for it is commendable in not a few that they will not enter those wide open gates until their preparation is such as would pass them freely through the most restricted way, nevertheless they are not exempt from the evil influence of those who do take advantage of this indiscriminate offer. Neither indeed, is any part of the profession free or unaffected by the ill results following in the wake of such a system of collegiate looseness. For at least the majority of those who enter without qualification do so with the view of getting out with their deplomas the easiest and quickest way, and as a matter of course, are ill prepared for a practice that would redound to the honor of their preciptor, the profession or their alma mater. To effect any change then for the better it is of the utmost importance that there be no clashing of demands upon the part of preceptors and colleges, and with reference to the student's acquirements. Furthermore, and that the foregoing may be encouraged, let the colleges require matriculants to pass a preliminary examination before receiving them, and let this examination be no mere matter of form, but let it bereal and earnest rigid thorough and without discrimina tion. By the college and profession thus working in unison, young men entering upon the study of dentistry, will be given to understand that there will be no royal road to preferment, neither open portals or even gates ajar by or through which they may pass, exept they be properly prepared.

Need I add that the office is the most suitable place for preparation in rudimentary details rather than the college, Possibly it might be urged as it had been by a few representatives of dental schools, that when matriculants were deficient, such deficiency must be compensated for by a closer application to studies within the college, and that after all no consequence could attach to imperfect acquisitions for a course of lectures, but that everything depended upon acquirements of a certain character and certain amounts of information previous to the students receiving certificates of graduation, this would undoubtedly suffice provided our colleges would not at times graduate men wholly undeserving such honors. Or it might obtain in the event that all such students were possessed of a desire to apply themselves with increased assiduity, and at the same time were clever enough to master in the limited time of college life the points of their insufficiency. But we can not flatter ourselves that this is true, neither can it be for the very intentions and incentives which impel such persons to accept or desire to accept generous offers at the hands of our colleges, are in most cases a complete bar to its consummation, and generally when diplomas are granted to such students, it is done with reserved protest, with a shrug of the shoulders and never without stultifying the noble efforts of those who have presented themselves fully abreast the mark, and are deserving their diplomas with every honor thereby conferred. Before concluding this part of our subject, we do not desire to leave the impression that all our institutions of learning or even all official representatives of any one of these, are indifferent regarding these matters just referred to.

For we can not doubt the honest intentions and ability of somemen connected with even the most indifferent colleges, but we do believe that honesty of purpose in one person is often under the swaying influence of dishonesty or petty jealousy in another, and for the want of independence suffer many violations of rules to go by without a word of censure. Then let us by all means have an understanding with reference to the question as to whether matriculants will be required to possess definite qualifications or not. If we can rely on our colleges imposing restrictions of such a nature and enforcing them without fear or favor, then indeed may we hope for a better state of things, and be encouraged to labor more earnestly in behalf of students under our care. Another plan of the subject which we are assured deserves a more hearty consideration than has been devoted to it, demands from us at least a passing notice. We refer to dental education for the masses as previously stated in this paper, popular education is one of the means by which students in dentistry, may be forced to acquire a thorough knowledge of their duties, and if anything has heretofore been left undone which to-day is in our power to accomplish, in this direction it surely behooves us as men desiring the promotion of our every interest to leave not a stone unturned, but to persevere in this as well as in other labors until what we desire is accomplished.

It is true that efforts upon the part of dental associations and individual practitioners have been made looking to this end; these have consisted principally of instruction given to patients during the very limited time they were under the dentist's care, and of small pamphlets published under the authority of societies or by individual dentists and distributed, to be in turn redistributed to the public. That in this way much good has been done, no one is foolish enough to doubt; but in many instances pamphlets sent out among our patients are carelessly tossed aside, and often never reach the ones who need their instructions the most.

What we desire and that which we consider our duty more than any one thing else towards the public is that information concerning these things should be secured to the young and rising generation and especially to those who are seldom, if ever under the care of competent practitioners. We are of the opinion that there is yet a more promising way of furnishing all necessary information to the popular mind upon this subject, than has yet been in operation. It will necessarily require much time and exertion, and may be accompanied with some considerable expense, but we think it is the only true method of solving this part of the problem and if so, is evidently worthy of all the requirements for its speedy introduction. An article published in the February number of the REGISTER and from our friend Prof. Chase of St. Louis, informs us that in twenty years from this time we will be in need of no more dentists in this country than are now practicing, all of which will occur as the result of so much hygienic advice. If this be true it is of grand significance and even if it be self abnegation, it is of that glorious sort which enables others to be happy without lessening materially our own happiness.

Many years ago a Dr. Cutter wrote in simple form and had published a treatise upon the subject of anatomy, physiology, and hygiene. This little work was introduced into many of the public and private schools in different portions of this country, and to its instructions many thousands are indebted for health and strength, and a knowledge of themselves such as enabled them to treat intelligently ordinary ailments and warned them in time when it became necessary to seek the assistance of physicians. Without commenting further upon the happy results which have followed the introduction of the books into our common schools, we will proceed to state that it is in the same manner we would propose to open the way for a more intelligent understanding of the teeth and their care by the masses. A book or books written in a simple though instructive form, and treating of the development and hygienic measures necessary to the health and preservation of the teeth, should be introduced at least in all our public schools and into as many private schools as possible. Seed of this character sown now in the fallow ground of our common schools will ere long spring up into bountiful harvest, and lighten the burdens of humanity throughout all time. And now young men a brief conclusion. This night ends your career as students in the Ohio College of Dental Surgery, these diplomas have been whispering all along the weary way, attain to the merit of this reward, and perfection will not be wanting; but do not flatter yourselves in this hour of long sought pleasure that the gleaming goal of perfection has been reached. No, not yet, thus far you have run well, and your bright faces leave no doubt of your happiness, but you are only catching the faint gleams of that shining mark, whose rays will continue to grow bright and yet brighter, ever lighting up more and still more the pathway of your future career.

College life is far too short to perfect you even in a specialty of a specialty, and if you would aim for the mastery of dentistry; if you would pursue each study in the college curriculum to the fullest extent that its application could be made subservient to your interests therein, how incomparably short seems the time of a collegiate life for such consu·mation. You have now arrived at that period of educational life, from which you can look back and recognize the fact that a college is not so much a place of learning, as it is a means of instructing you how to learn.

Joseph Cook, in one of his inimitable lectures tells us that faithfulness to all college studies, sends one into the brown wheat fields at last with reaping machines of the first order.

And now let me ask; are your diplomas symbols of faithfulness to studies? I have every reason for believing they are, and take it for granted that you are fully prepared for the labors before you. The sickle is sharpened, the gearing is adjusted and runs smoothly, and you stand there equipped for the broad never ending harvest field, whose good grain and cheat grow side by side. See to it then when you come to garner the results of each days labor that no cheat is there to blacken or contaminate the field you sow again.

To-night you shake off the manacles of servitude within these walls, to-morrow you will be bound again in the interests of your profession and common humanity.

With the morning sun begins the duties of your new life; nerve yourselves for the conflict; be sure you start aright, and God bless the hearts that warm with good intentions, and weary not in carrying them out.

